# Remifentanil-Propofol-Ketamine- Based Total Intravenous Anesthesia with Spontaneous Breathing for Adult Rigid Bronchoscopy

**DOI:** 10.3390/jcm14020377

**Published:** 2025-01-09

**Authors:** Luca Frasca, Antonio Sarubbi, Filippo Longo, Fabio Costa, Domenico Sarubbi, Alessandro Strumia, Valentina Marziali, Pierfilippo Crucitti

**Affiliations:** 1 Department of Thoracic Surgery, Fondazione Policlinico Universitario Campus Bio-Medico, Via Alvaro del Portillo, 200, 00128 Rome, Italy; l.frasca@policlinicocampus.it (L.F.); filippo.longo@policlinicocampus.it (F.L.); p.crucitti@policlinicocampus.it (P.C.); 2 PhD Course in Microbiology, Immunology, Infectious Diseases, and Transplants (MIMIT), University Tor Vergata, Viale Oxford, 81, 00133 Rome, Italy; 3 Master’s Degree Program in Medicine and Surgery, Campus Bio-Medico University, Via Alvaro del Portillo, 21, 00128 Rome, Italy; 4 Operative Research Unit of Anesthesia and Intensive Care, Fondazione Policlinico Universitario Campus Bio-Medico, Via Alvaro del Portillo, 200, 00128 Rome, Italy; f.costa@policlinicocampus.it (F.C.); d.sarubbi@policlinicocampus.it (D.S.); a.strumia@policlinicocampus.it (A.S.)

**Keywords:** rigid bronchoscopy, central airway obstruction, anesthetic management, spontaneous assisted ventilation, airway stenting

## Abstract

**Background:** Rigid bronchoscopy (RB) is the gold standard for managing central airway obstruction (CAO), a life-threatening condition caused by both malignant and benign etiologies. Anesthetic management is challenging as it requires balancing deep sedation with maintaining spontaneous breathing to avoid airway collapse. There is no consensus on the optimal anesthetic approach, with options including general anesthesia with neuromuscular blockers or spontaneous assisted ventilation (SAV). **Methods:** This case series presents our anesthetic protocol using remifentanil–propofol–ketamine total intravenous anesthesia (TIVA) with SAV in four patients with airway obstructions. Muscle relaxants were avoided in all cases. **Results:** Ketamine’s ability to preserve respiratory drive and airway reflexes, along with its bronchodilating properties, made it ideal for managing CAO. All procedures successfully restored airway patency without complications or drug-related side effects. **Conclusions:** Our findings suggest that remifentanil–propofol–ketamine TIVA combined with SAV is a viable anesthetic approach for therapeutic RB, offering effective sedation, maintaining airway patency, and minimizing perioperative complications.

## 1. Introduction

Rigid bronchoscopy (RB) is the gold standard for the management of central airway obstruction (CAO), a potentially life-threatening condition [1]. Malignant causes are more common, largely due to the increasing incidence of primary lung cancer [2]. Non-malignant CAO is most frequently caused by iatrogenic injury and connective tissue disorders [2]. Interventional techniques for CAO, such as laser ablation, argon plasma coagulation (APC), and airway stenting, are crucial in maintaining airway patency [3].

RB, despite its effectiveness, is a high-risk procedure, posing challenges for anesthetic and airway management [4]. There is no consensus on the optimal anesthetic approach for rigid bronchoscopy in adults with CAO, with options including general anesthesia with neuromuscular blockers (NMBs) or spontaneous assisted ventilation (SAV) [5]. The use of muscle relaxants results in loss of airway patency due to central airway collapse, complicating ventilation due to limited airway access for the anesthesiologist [4,6]. In contrast, SAV without NMB agents may reduce the risk of severe hypoxemia but requires continuous adjustments of anesthesia depth to ensure patient safety and comfort during therapeutic RB [1,5,7].

Remifentanil and propofol remain the mainstay drugs for TIVA due to their potent and short-acting properties [1]. Nevertheless, the combination of an opioid with propofol is associated with several side effects, such as respiratory depression, postoperative nausea and vomiting, propofol-induced hypotension, and opioid-induced bradycardia. On the other hand, there is growing interest regarding the use for ketamine for endobronchial procedures [8]. Ketamine is occasionally used as an adjunct to propofol in TIVA regimens due to its unique pharmacological properties [9]. It induces a dissociative state with analgesic, sedative, and amnesic effects, while maintaining respiratory drive and airway reflexes thus offering the advantage of avoiding respiratory or cardiovascular depression [7,10].

Although ketamine is commonly employed in airway surgery, evidence on bolus infusions of ketamine and propofol as adjuncts to remifentanil-based anesthesia for RB in adults remains limited.

We present our anesthetic protocol based on remifentanil–propofol–ketamine TIVA and SAV for RB-guided tracheobronchial stenting in four patients with CAO at a single institution.

## 2. Materials and Methods

We performed rigid bronchoscopic intervention in four patients with CAO. Three of them were affected by lung cancer and one patient suffered from benign post intubation tracheal stenosis. We describe our procedural and anesthesia-related outcomes. Patient characteristics and procedural details are listed in Table 1 and Table 2.

Written informed patient consent for the publication of this case series was obtained from all patients, and the manuscript adheres to Enhancing the Quality and Transparency of Research (EQUATOR) guidelines.

Topical anesthetics reduced the need for sedatives in RB, including 0.5% ropivacaine (25 mg), dexamethasone (4 mg), and 2% nebulized lidocaine 2% + 10 mcg dexmedetomidine (DXM) via an intranasal atomizer [11]. All patients also received 20 mg IV dexamethasone and 0.5 mg atropine to prevent edema and reduce secretions. Thereafter, analgosedation with midazolam 20–40 mcg/kg and fentanyl 1 mg/kg was administered as premedication to alleviate patient discomfort.

**Figure 1:**
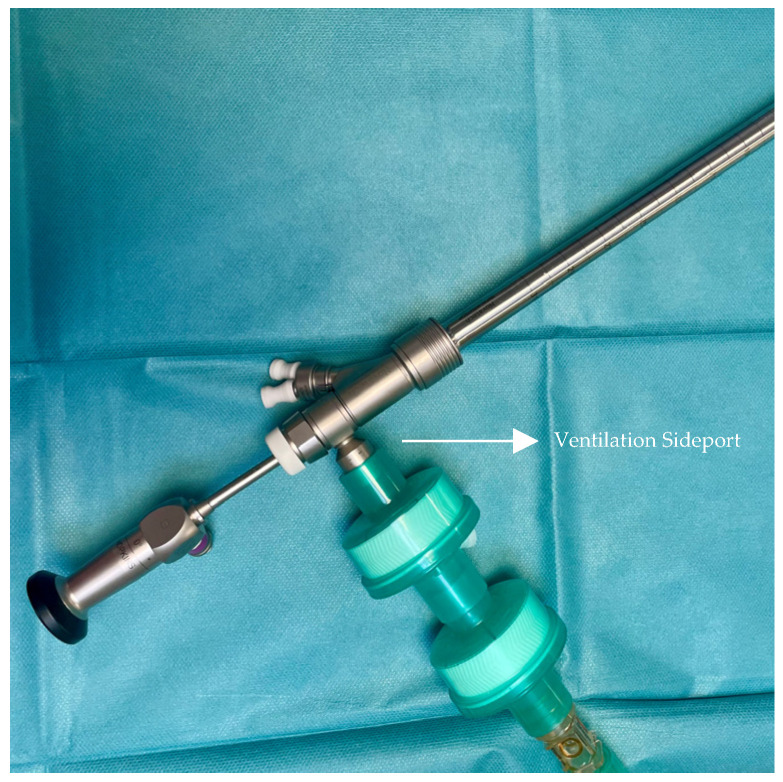
Ventilation through the external side port of the rigid bronchoscope.

Bronchoscopy was performed in the emergency operating room. The operative report and anesthetic record from each procedure was reviewed and details of the procedure, anesthetic approach, intraoperative hemodynamic and respiratory parameters, and postoperative outcomes were collected. All patients were monitored with electrocardiograph (EKG), pulse oxygen saturation (SpO_2_) and Bispectral Index (BIS). Noninvasive blood pressure (NIBP) was measured in 5 min intervals during RB. The respiratory circuit of the anesthesia machine (Dräger Perseus^®^ A500, Lübeck, Germany) remained continuously connected to the lateral ventilation port of the bronchoscope, allowing patients to breathe through it (Figure 1). Set to manual/spontaneous mode, the machine had the adjustable pressure-limiting valve fully open, fresh gas flow at maximum (15 L/min), and an O_2_ concentration of 50–80%. This setup enabled spontaneous breathing support, with options for manual or mechanical assistance, as well as continuous positive airway pressure (CPAP) if needed. High fresh gas flow and leaks from the open proximal end of the bronchoscope compromised accurate measurements of respiratory rate (RR) and end-tidal (ET) CO_2_, resulting in unreliable data; thus, these parameters were not reported

Case 1 

**Figure 2:**
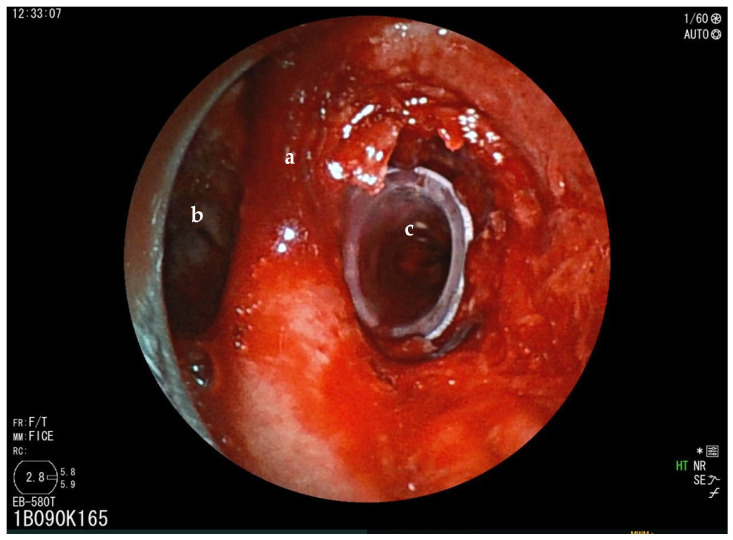
Rigid bronchoscopy of case 1 (a = carina; b = left main bronchus). Bronchoscopic view immediately after dilation and then stenting with bronchial silicon stent. Lumen of right stem bronchus is now completely open (c).

The first patient was a 75-year-old male with squamous cell carcinoma (SCC) involving the right lung and causing complete obstruction of the right stem bronchus. After preoxygenation for 5 min with 100% oxygen, TIVA was started with remifentanil target-controlled infusion (TCI) 0.5–3 ng/mL and ketamine (0.75–1 ng/kg). For the latter, an initial dosage of 50 mg was administered, followed by additional doses of ketamine (20 mg) and propofol (TCI 0.5–1.5 ng/mL) every 20–40 min until the end of the procedure. After intubation with the rigid bronchoscope, spontaneous breathing was maintained throughout the procedure. The patient remained hemodynamically stable throughout the procedure. An episode of oxygen saturation below 90% occurred; ventilation was assisted manually and oxygen levels promptly restored. APC allowed debulking of the tumor to achieve the desired patency. Thereafter, successful placement of a NOVATECH^®^ GSS^TM^ (La Ciotat, France) bronchial silicon stent was performed to re-establish airway patency (Figure 2).

The procedure concluded in 120 min and the patient was then transferred to the intensive care unit (ICU) for observation. The patient was discharged from the hospital after 5 days and he was able to maintain a satisfactory life without dyspnea for 3 months thereafter.

Case 2

**Figure 3:**
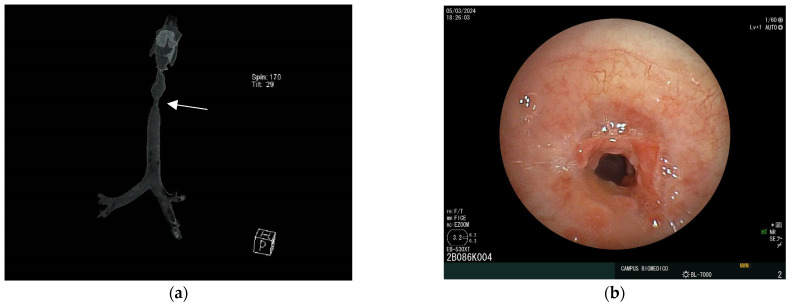
Benign subglottic stenosis secondary to traumatic intubation treated by sequential rigid dilations. (**a**) Reconstruction of CT scan of trachea showing mid-tracheal stenosis (arrow pointing at narrowest caliber); (**b**) bronchoscopic images showing post intubation scar with stenosis of tracheal lumen.

Case 2 is a 24-year-old obese (BMI 34.89) female patient who developed a fibrotic mid-tracheal complex stenosis after urgent 4-day intubation due to a seizure episode. After a month of extubation, the patient exhibited severe stridor, dyspnea, and tirage. Anesthesia was provided using the identical drug and dose regimen as in case 1. Even incomplete, the mechanical dilation of the stenosis with the rigid bronchoscope was immediate (Figure 3). Given the complex stenosis, a NOVATECH^®^ GSS^TM^ BD standard tracheal silicone stent was placed through the rigid bronchoscope to achieve the desired patency and to prevent disease recurrence. HR (80–90 bpm) and BP (110/70 mmHg) were stable and arterial oxygen saturation (SaO_2_) was maintained around 88–95% during the procedure. The patient tolerated the procedure without complications and noted immediate improvement in her respiratory status. She was discharged from the hospital after 4 days and required no further airway procedures.

Case 3

Case 3 is a 77-year-old male with advanced lung adenocarcinoma presented with dyspnea and hypoxia caused by complete obstruction of the right main bronchus. The anesthesia drug regimen included IV remifentanil and ketamine (identical dosage as in case 1). In this case, instead of propofol, a continuous infusion of DXM 0.7–1.4 μg/kg/h was administered to maintain intraoperative depth of anesthesia. RB started after the patients had no obvious physical activity. The rigid bronchoscope was advanced gently into the stenosis and APC was used as a method to desiccate and coagulate the tumor. Despite mechanical debulking of the endobronchial lesion, there was still 50% obstruction of the trachea. The decision was made to place a NOVATECH^®^ GSS^TM^ BD bronchial silicon stent to re-establish airway patency. Despite DXM infusion, the patient remained hemodynamically stable, probably thanks to the ketamine infusion. SaO_2_ was maintained at 92–100% during the procedure. The intervention was tolerated without further distress or complications. The patient’s main symptoms were relieved; however, he died two weeks later of disease progression.

Case 4

Case 4 is a 74-year-old female with lung SCC who presented with a two-week history of dyspnea, cough, solid food dysphagia, and weight loss. The patient had a subcarinal tracheal mass invading into the carina and lower portion of the trachea causing approximately 60% obstruction. Anesthesia was induced and maintained with the same regimen as case 1 with IV propofol, remifentanil, and ketamine. With the patient still breathing spontaneously, rigid dilation and tumor debulking with APC were performed. After dilation to desired patency, the bronchoscope was positioned at the estimated distal end of the stent. The scope was then retracted to the desired proximal section of the stent by grasping the end of the rigid barrel. A customized Merit Medical Systems AERO^®^ (South Salt Lake, UT, USA) 40 mm metallic stent was placed in the trachea and main stem bronchi. The patient had good hemodynamics (HR: 70–80 bpm; BP: 110/75 mmHg) and oxygen saturation (89–100%) during the procedure. The patient’s respiratory condition strongly improved immediately after the procedure. However, she died at the hospital one month later of disease progression.

## 3. Discussion

RB is the gold standard for managing CAO, a potentially life-threatening condition that can arise from both malignant and benign causes [12]. Currently, airway stenting through RB typically requires deep sedation or even general anesthesia [12]. Neuromuscular blockade is not an absolute requirement and there are some debates whether the use of paralytic agents is safe and indispensable in this procedure [4]. The use of NMBs abolishes muscle tone, potentially leading to complete obstruction and loss of airway patency [1,6]. In this process, multiple ventilation strategies through an open circuit can be considered including apneic oxygenation, SAV, controlled ventilation, and jet ventilation [4,6]. SAV is a ventilation technique in which sedation levels are closely titrated during the procedure to maintain spontaneous breathing by the patient [6]. Specifically, SAV may have a lower rate of post-procedural reintubation due to the avoidance of NMB agents [6].

As described in patient 1, in the case of oxygen desaturation, assisted or controlled ventilation can be achieved by removing instruments from the bronchoscope, attaching the ventilator circuit adapter to its distal end, and using the rigid scope as an endotracheal tube [2]. This system allows the delivery of positive pressure breaths and positive end-expiratory pressure (PEEP), improving ventilation and oxygenation [2]. In the same case, we applied a moderate pressure support of 10 cm H_2_O to assist spontaneous ventilation. Mouth packing was not required as air leakage during SAV did not affect oxygenation, and the brief period of pressure support ventilation did not compromise gas exchange. As stated above, data on ET-CO_2_ and RR were not reported because they were unreliable due to an unsealed circuit; however, continuous monitoring of respiratory movements, chest expansion, and pulse oximetry allowed us to detect any respiratory changes.

Since SAV proved sufficient to effectively maintain gas exchange, we were able to avoid the use of jet ventilation in all cases. This helped prevent potential complications such as hypercapnia, barotrauma with air trapping, tension pneumothorax, and subcutaneous emphysema [6]. On the other hand, using SAV without NMB requires the anesthesiologist to tailor the administration of analgesic and hypnotic agents [6]. Murgo et al. showed that RB can be safely conducted using a combination of TIVA and SAV [5]. Accordingly, we employed a remifentanil–propofol–ketamine-based TIVA with SAV, avoiding NMBs in all cases. Generally a combination of propofol and short-acting opioids is administered simultaneously [2]. Propofol and remifentanil-based TIVA is preferred over inhalational anesthesia to prevent environmental leakage from the unsealed respiratory circuit [7].

The pharmacological properties and clinical application of ketamine, especially for sedation for flexible bronchoscopic procedures, were already investigated [6,13]. This medication has bronchodilating properties and it does not cause respiratory or cardiovascular depression [9]. In our center, similar procedures without ketamine have traditionally been managed with general anesthesia, using propofol and remifentanil for sedation and analgesia, along with muscle relaxation. 

Despite ketamine use, the remifentanil TCI rate had to be gradually adjusted to standard levels (1.5–3 ng/mL) to control hemodynamic responses. Without ketamine, higher remifentanil doses may have been required, potentially suppressing respiratory drive and leading to respiratory depression or apnea. Therefore, adding ketamine to TIVA regimens has a synergistic pharmacodynamics impact that lowers the need for opioids [14]. Additionally, increasing the risk of postoperative hyperalgesia was prevented by intraoperative ketamine infusion, suggesting a role for the N-methyl-D-aspartate pain-facilitation process [15].

On the other hand, ketamine sedation has some side effects. While BIS was used, its accuracy may be compromised due to ketamine’s known effects on readings. Although controversial, BIS monitoring remains useful in minimizing the risk of respiratory depression from remifentanil overdose and reducing reflex movement [1,5]. Moreover, ketamine increases arterial BP, HR, and cardiac output [1]. Indeed, its hypertensive effects may elevate the risk of bleeding during the debulking process through APC. Both propofol and midazolam mitigate ketamine’s sympathomimetic effects through CNS inhibition, achieving a balanced sedative state during procedures [10]. Additionally, ketamine should not be administered alone because it can lead to significant dissociative effects and hallucinations, especially in the elderly. [16]. In a surgical setting, ketamine is typically combined with benzodiazepines to mitigate these adverse psychological symptoms [16]. Finally, ketamine has the potential to increase postoperative nausea and vomiting (PONV), but no episodes were observed among our cases [9]. In our experience, considering the life-saving nature of the procedure, we determined that maintaining hemodynamic stability clearly outweighed these potential complications.

In patient 3, as an alternative to propofol, we administered DXM to maintain patient compliance. DXM, as an alpha2 receptor agonist, carries bradycardia and hypotension as common side effects. The combination of DXM and ketamine seems to provide adequate sedation mitigating hemodynamic changes related to both drugs administered singularly [7,12,17]. Beside sedation and ventilation, topical anesthesia has a major role in this kind of procedure. In the described cases, we adapted the recent findings on local anesthetic mixtures and adjuncts and used a ropivacaine–dexamethasone–DXM mixture, which ensured effective analgesia and minimized coughing, with no additional pain medication needed in the first 24 h [18].

The insertion of fixed-diameter metallic and silicone stents through RB provides immediate palliation of symptoms in patients with CAO and severe breathing difficulties [17]. Our stents, including the NOVATECH^®^ silicone stents and AERO^®^ 40 mm fully covered metallic stents, provided immediate relief of symptoms, even in complex and advanced disease scenarios. Complications related to RB may be classified as pre-operative, anesthesia-associated, or due to the procedure itself. Careful planning and coordination by experienced surgical and anesthetic teams is essential [6,19].

## 4. Conclusions

Our series showed that remifentanil–propofol–ketamine-based TIVA is a feasible and beneficial approach for therapeutic RB. Nevertheless, the limited sample size restricts the generalizability of our results, necessitating cautious interpretation. Randomized studies are needed to validate the safety, optimal dosages, and efficacy of this approach in high-risk procedures.

## Figures and Tables

**Table 1:** Demographics and clinical characteristics.

	Age (Years)/Sex	Smoking History	ASA-PS Classification	Comorbidities	Preop Respiratory Status	Etiology	Histology	Location
Patient 1	75/Male	Past	III	COPD, Hypertension	Dyspnea	Malignant	Squamous NSCLC	Right main bronchus
Patient 2	24/Female	Never	II	Obesity	Dyspnea and stridor	Benign		Laryngotracheal
Patient 3	77/Male	Past	III	Hypertension	Dyspnea, hypoxia	Malignant	Adenocarcinoma NSCLC	Right main bronchus
Patient 4	74/Female	Past	IV	None	Dyspnea, cough, dysphagia, weight loss	Malignant	Squamous NSCLC	Carina

ASA-PS: the American Society of Anesthesiologists—Physical Status (ASA II/III: patient with mild/severe systemic disease; ASA IV: patient with severe systemic disease that is constant threat to life); NSCLC: non-small cell lung cancer.

**Table 2:** Procedural details, complications, and outcomes.

	Duration of Procedure (min)	Ablative Technique	Stent Placed	Anesthesia	Modes of Ventilation	Procedural Complications	Discharge (Days)	Mortality (Days)
Patient 1	120	APC	NOVATECH, La Ciotat, France^®^ bronchial silicon	TIVA	SAV	Stent positioning	5	120
Patient 2	70	None	NOVATECH, La Ciotat, France^®^ tracheal silicon	TIVA	SAV	None	4	Alive
Patient 3	80	APC	NOVATECH, La Ciotat, France^®^ bronchial silicon	TIVA	SAV	None	6	60
Patient 4	95	APC	AERO^TM^, South Salt Lake, UT, USA 40 mm	TIVA	SAV	Stent positioning	Death at the hospital	25

APC: argon plasma coagulation; TIVA: total intravenous anesthesia; SAV: spontaneous-assisted ventilation.

## Data Availability

The datasets generated and/or analyzed during the current study are available from the corresponding author on reasonable request.
